# Colonization Priority of Spider Mites Modulates Antioxidant Defense of Bean Plants

**DOI:** 10.3390/insects17020145

**Published:** 2026-01-27

**Authors:** Tairis Da-Costa, Julia Renata Schneider, Aline Marjana Pavan, Luana Fabrina Rodighero, Anderson de Azevedo Meira, Noeli Juarez Ferla, Geraldo Luiz Gonçalves Soares

**Affiliations:** 1 Laboratório de Evolução, Ecologia Química e Quimiotaxonomia, Instituto de Biociências, Universidade Federal do Rio Grande do Sul, Porto Alegre 91501-970, Brazil; 2 Laboratório de Acarologia, Tecnovates, Universidade do Vale do Taquari—Univates, Lajeado 95913-528, Brazil

**Keywords:** herbivores, population growth, *Tetranychus ludeni*, *Tetranychus urticae*

## Abstract

The first species of herbivore to colonize the plant can obtain advantages and displace other organisms to less favorable substrates. This study evaluated whether the colonization order of the herbivores *Tetranychus ludeni* Zacher and *T. urticae* Koch (Tetranychidae) influences their population dynamics and antioxidant enzymatic activity of bean plants (*Phaseoulus vulgaris* L.). Experiments manipulating the colonization priority were performed, and the activities of the enzymes ascorbate peroxidase (APX), catalase (CAT), and superoxide dismutase (SOD) were evaluated. The results indicated priority for *T. urticae* led to a significant reduction in *T. ludeni* abundance, suggesting that the order of species influences the total abundance of mites. The presence of mites affected the antioxidant enzymatic activities APX, CAT, and SOD. These findings provide important insights into the response of bean plants to herbivorous mite infestation, highlighting the role in plant defense against *T. ludeni* and *T. urticae*.

## Simple Summary

This study investigated how the order of colonization by two herbivorous mite species influences their abundance and the antioxidant enzyme activity in bean plants (*Phaseolus vulgaris* L.). The authors tested the hypothesis that the first herbivore species to colonize the plant gains an advantage over the other and examined how the host responds to herbivory. The results showed that initial colonization by *Tetranychus urticae* significantly reduced the abundance of *Tetranychus ludeni*. In addition, infestation induced changes in the enzymatic activity of bean plants, indicating the activation of plant defense mechanisms. It was also demonstrated that the order of mite colonization is a determinant factor in the interactions between these species and the host response. This information is relevant to agricultural systems and production, as it contributes to understanding the behavior of agricultural pests and plant responses to herbivory, supporting the development of sustainable and efficient integrated pest management strategies.

## 1. Introduction

The theory of modern coexistence defines that the survival of different species is made possible through two maintenance mechanisms: equalizers, which reduce the average competitive fitness, and stabilizers, which promote more intense competition within species than between them [1]. The coexistence of species is achieved when the differences in occupied niches are greater than the differences in their competitive capabilities; if the differences in fitness between competitors are more significant, in this way, the species with greater fitness predominates in the community [1,2,3].

According to the priority effect, the first herbivorous species that initially colonizes the plant can alter the host conditions, obtaining a competitive advantage and influencing the establishment of other herbivorous species that arrive later [4,5]. Therefore, by arriving early at the plant, the first herbivore may have benefits, for example, occupying its preferred niche and displacing the others to non-ideal substrates [6]. The colonization priority of mite species can impact their distribution on the plant and the activation or suppression of defenses in the presence of interspecific competitors [7,8]. *Tetranychus ludeni* Zacher and *Tetranychus urticae* (Koch) (Tetranychidae) coexist in many species of native and cultivated plants, such as bean (*Phaseolus vulgaris* L.), soybean (*Glycine max* L.) (Fabaceae), and tomato (*Solanum lycopersicum* L.) (Solanaceae) [9,10,11], and this sharing can influence intraspecific and interspecific interactions.

*Tetranychus urticae,* for example, induces defense in various plant species [12,13,14] and *T. ludeni* suppression defenses in tomato plants [7] and it can demonstrate a moderate oxidative stress response in soybean plants [15]. In this case, defenses may differ depending on the order of arrival of herbivores on the plant [16]. Additionally, by feeding on plants, these mites damage the leaf tissue, potentially leading to the accumulation of reactive oxygen species (ROS), triggering oxidative stress [17,18], leading to cellular toxicity [19,20].

To maintain redox homeostasis and ROS at basal levels, plants rely on an antioxidant defense system, composed of enzymes and non-enzymatic compounds [18]. The main antioxidant enzymes are ascorbate peroxidase (APX) (EC 1.11.1.11), catalase (CAT) (EC 1.11.1.6), and superoxide dismutase (SOD) (EC 1.15.1.1) [19,21]. APX has an essential role in protecting plant cells against environmental stresses, in addition to contributing to plant growth and development [22]. CAT is responsible for maintaining redox homeostasis by catalyzing the decomposition of hydrogen peroxide (H_2_O_2_) into water (H_2_O) [23,24]. SOD is the first enzyme activated in plants in response to stress factors [25,26]. These antioxidant enzymes can strengthen plant metabolism, providing tolerance to mites, which may hinder their establishment, although enzymatic responses depend on several factors, such as plant species, cultivar, mite species, toxins, and infestation time [27]. The enzymatic response of plants to mite infestation needs greater attention, as existing studies make it difficult to formulate clear and precise conclusions on this subject [27].

The objective of this study was to evaluate whether the colonization priority of the herbivores, *T. ludeni* and *T. urticae*, influences the population dynamics and the antioxidant enzymatic activities produced by the plant. Since priority effects can be mediated by the modulation of plant defenses, we hypothesize that colonization priority influences population dynamics, especially when *T. urticae* arrives first, harming the population development of *T. ludeni*. Furthermore, antioxidant enzymes are influenced by the coexistence of these two species in the plant.

## 2. Plant Cultivation

The *T. ludeni* population was established from specimens obtained from soybean leaves in the municipality of Muitos Capões, state of Rio Grande do Sul. The population of *T. urticae* was established from specimens collected from grapevine leaves in the municipality of Garibaldi, state of Rio Grande do Sul. The populations of each species were maintained on bean plants (*Phaseoulus vulgaris* L. *cv* Mouro) in a climate-controlled room at 26 ± 2 °C, with a 12 h light cycle and 70 ± 10% relative humidity (RH). The colonies had been maintained under laboratory conditions for one year.

For the experiments, four bean seeds of the same cultivar were sown in plastic pots (14 × 10 cm), with commercial substrate (Carolina Soil, Carolina Soil, Santa Cruz do Sul, Brazil) and kept in a climate-controlled room (26 ± 1 °C; 12 h of photophase and 70 ± 5% RH). After seven days, only one plant/pot was maintained, a wooden rod was added close to the stem to aid support and the mites were released.

### 2.1. Population Experiments

To evaluate the effect of the colonization priority of herbivores on population dynamics, experiments were used to manipulate the colonization priority of mites. For this, five treatments were performed and the number of individuals of each species is shown in parentheses: Treatment 1 (T1): *T. ludeni* alone (20♀); Treatment 2 (T2): *T. urticae* alone (20♀); Treatment 3 (T3): *T. ludeni* and *T. urticae* together (10♀:10♀); Treatment 4 (T4): *T. ludeni* (1st) released four days before *T. urticae* (2nd) (10♀:10♀); Treatment 5 (T5): *T. urticae* (1st) released four days before *T. ludeni* (2nd) (10♀:10♀). The treatments were replicated ten times, i.e., ten plant pots/treatment.

Mated females, two days old, were transferred to bean leaves using a fine-tipped brush. The plants were seven days after emergence and had two leaves. Females of each species were counted under a binocular stereomicroscope 14 days after infestation, as this is the time for a new generation. Eggs and juveniles were not counted because the species are very similar in the immature stages. Adults of *T. ludeni* are bright red in color, while *T. urticae* has two dark spots on its body, which makes it easy to distinguish between adults of the two species.

### 2.2. Antioxidant Enzyme Activity

To evaluate the plant’s antioxidant defense metabolism, we selected three key antioxidant enzymes: SOD, the front line of defense that acts against superoxide, and two peroxidases: CAT and APX, which act on hydrogen peroxide. To assess antioxidant enzyme activity, the last trifoliate leaves (the youngest leaves) produced by the plant were collected, weighed, and frozen in liquid nitrogen. This material was macerated with mortar and pestle, with liquid nitrogen, homogenized with four times its mass in a buffer solution (170 mM sodium chloride, 3 mM potassium chloride, 5 mM disodium phosphate, and 2 mM monopotassium phosphate), centrifuged (2000 force- G), for ten minutes, at 4 °C (refrigerated L-3 Loccus microtube centrifuge). The supernatant was pipetted and stored at −20 °C until analysis.

The determination of total soluble proteins (mg/mL) used the Comassie method [28]. In test tubes, 100 µL of enzymatic extract was added, followed by 900 µL of Comassie. The mixture was vortexed for 10 s, incubated in the dark for 10 min, and then placed in a 1 mL cuvette for absorbance reading using a spectrophotometer (Shimadzu UV-1800, Shimadzu, Kyoto, Japan) at a wavelength of 595 nm. The values were calculated based on the equation obtained from a standard curve with known concentrations of albumin.

APX activity was quantified using the Nakano and Asada [29] method. Analysis was performed with duplicates and a reaction mixture was prepared with potassium phosphate buffer (50 mM and pH 6.8), hydrogen peroxide (1 mM), and ascorbate (0.8 mM). In 3 mL quartz cuvettes, 1950 µL of the reaction mixture and 50 µL of extract were added, homogenized, and the absorbance was read on a spectrophotometer (Shimadzu UV-1800, Shimadzu, Kyoto, Japan), wavelength of 290 nm, for five minutes, every twenty seconds. The enzyme activity was calculated based on the ascorbate oxidation capacity in one minute, considering the extinction coefficient of 2.8 mM/cm [30].

CAT had its activity determined by the method of Cakmak and Marschner [31]. This method evaluates enzyme activity based on the ability to metabolize H_2_O_2_. The analysis was performed in duplicate, and a reaction mixture was prepared with potassium phosphate buffer (50 mM and pH 6.8) and H_2_O_2_ (20 mM). In 3 mL quartz cuvettes, 1950 µL of the reaction mixture was homogenized with 50 µL of the enzymatic extract and the absorbance was measured in a spectrophotometer (Shimadzu UV-1800, Shimadzu, Kyoto, Japan) at 240 nm, for five minutes, every twenty seconds. For the calculation, the extinction coefficient of 36 M/cm was considered [30].

SOD was determined by the enzyme’s ability to reduce nitro-tetrazolium blue, photochemically, as proposed by Del Longo et al. [32], where one unit of SOD is defined as the amount of enzyme necessary to inhibit the photoreduction of nitrotetrazolium blue by 50% [33]. The analysis was performed in duplicates, and a reaction mixture was prepared with potassium phosphate buffer (50 mM and pH 7.8), methionine (13 mM), nitrotetrazolium blue (75 µM), ethylenediaminetetraacetic acid (EDTA, 0.1 mM), and riboflavin (2 µM). To 1 mL cuvettes, 960 µL of this mixture was added and homogenized with 40 µL of enzymatic extract. These samples were incubated at 25 °C, exposed to a 15 W lamp for 10 min. After this period, a spot reading was carried out on a spectrophotometer (Shimadzu UV-1800, Shimadzu, Kyoto, Japan), at a wavelength of 560 nm.

According to the developers of the method, Giannopolitis and Ries [34], this reading allows recording the production of formazan blue, which results from the photorecovery of nitrotetrazolium blue. The calculation of SOD activity (SOD unit/mg protein) took into account the difference between the white, light, and dark readings, that is, one kept under light with the samples, and the other incubated for the same period in the dark.

### 2.3. Data Analyzes

To test whether *T. ludeni* had a higher abundance when it arrives first and whether there is any shift to one of the leaf regions (abaxial/adaxial) when both species are together, generalized linear mixed effects models (GLMM) were fitted. Considering that the number of mites colonizing the plants did not adequately fit the models with data normality assumptions as it is not a type of continuous variable, two different GLMMs were adjusted for subsequent model selection. Thus, a GLMM with a Poisson distribution family and another with a negative binomial distribution were adjusted.

Both GLMMs were structured as follows: the response variable was the number of mites on the plants, while the predictor variables were the type of treatment (T1–T5) and leaf region (abaxial/adaxial). Both GLMMs were performed using the lme4 package [35] with the function glmer for Poisson and glmer.nb for negative binomial. A GLMM with negative binomial is an alternative model to Poisson when the latter presents overfitting problems. To select the model that best fit the data, the AICctab function from the bbmle package was used [36,37].

The results and parameters of the selected model were extracted using the Anova function from the car package [38]. Finally, we used the lsmeans and pwpm functions from the emmeans package [39] for pairwise comparisons. The *p*-values of these pairwise comparisons were adjusted using the False Discovery Rate (FDR) method, available in the emmeans package [39]. All analyzes were performed in the R software [37,40].

To evaluate the activities of antioxidant enzymes, the normality of residuals was verified using the Shapiro–Wilk test. The data were subjected to Analysis of Variance (ANOVA) and the means were compared with Tukey’s test at 5% significance, in the R software [37]. Enzyme activity data and mite quantities were correlated by Pearson in the corrplot package of the R software [37].

## 3. Results

The results found indicate significant effects of treatments on mite populations (χ^2^ = 320.94; df = 7; *p* < 0.001; Table 1), suggesting that the colonization priority of the species significantly influenced the acarine abundance in the plant.

**Figure 1:**
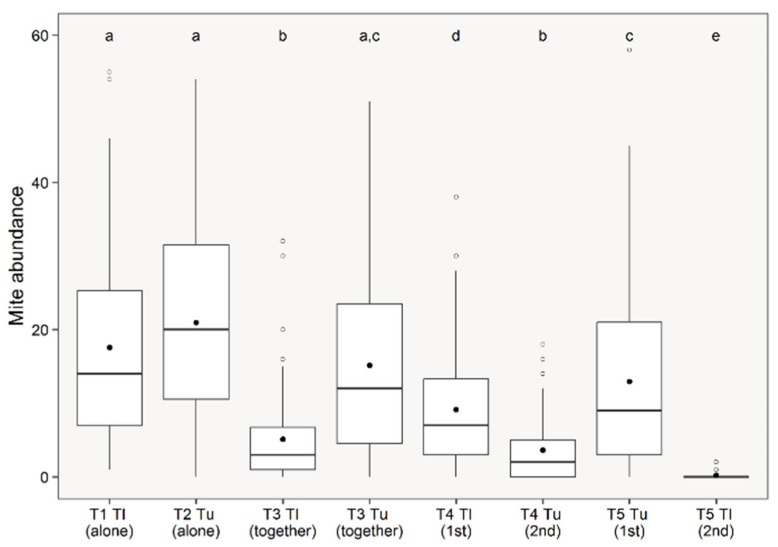
Paired comparisons between treatments in relation to the number of *Tetranychus ludeni* and *Tetranychus urticae* released individually, simultaneously or after four days on bean plants. Notes = boxplots: box − interquartile range (IQR) covering the 2nd and 3rd quartiles, inner line = median, whiskers = data dispersion, outliers = open points, means = closed black points. Letters above boxplots indicate significant differences if they are distinct among every pair group. (T1: *T. ludeni* alone; T2: *T. urticae* alone; T3: *T. ludeni* and *T. urticae* together; T4: *T. ludeni* (1st) and *T. urticae* (2nd); T5: *T. urticae* (1st) and *T. ludeni* (2nd)).

Considering pairwise comparisons, most treatments differed significantly, with few exceptions (Table 1; Figure 1). T1 and T2 did not show a significant difference (GLMM, z-value = −0.70, *p* = 0.484), suggesting similar colonization patterns between *T. ludeni* and *T. urticae* when released alone on bean plants. However, when comparing T1 and T3, T1 had a greater abundance of *T. ludeni* than T3 (GLMM, z-value = 8.34, *p* < 0.001), indicating an advantage when alone on the plant. Likewise, when comparing T1 and T4, T1 had a larger population than T4, for *T. ludeni* (GLMM, z-value = 4.73, *p* < 0.001). Between T1 and T5, T1 had a higher population of *T. ludeni* than T5 (GLMM, z-value = 12.51, *p* < 0.001).

When comparing the *T. urticae* population, T2 and T3 there was no difference (GLMM, z-value = 2.40, *p* = 0.020) (Table 1; Figure 1). When comparing *T. urticae* between T2 and T4, T2 had a larger population than T4 (GLMM, z-value = 10.18, *p* < 0.001); between T2 and T5, T2 also had a higher *T. urticae* population than T5 (GLMM, z-value = 4.03, *p* < 0.001).

However, *T. urticae*, even with the presence of *T. ludeni*, is able to establish itself and have a higher population growth, similar to when it is alone on the plant. *Tetranychus ludeni* presents development difficulties when it is with *T. urticae*, in both treatments.

**Figure 2:**
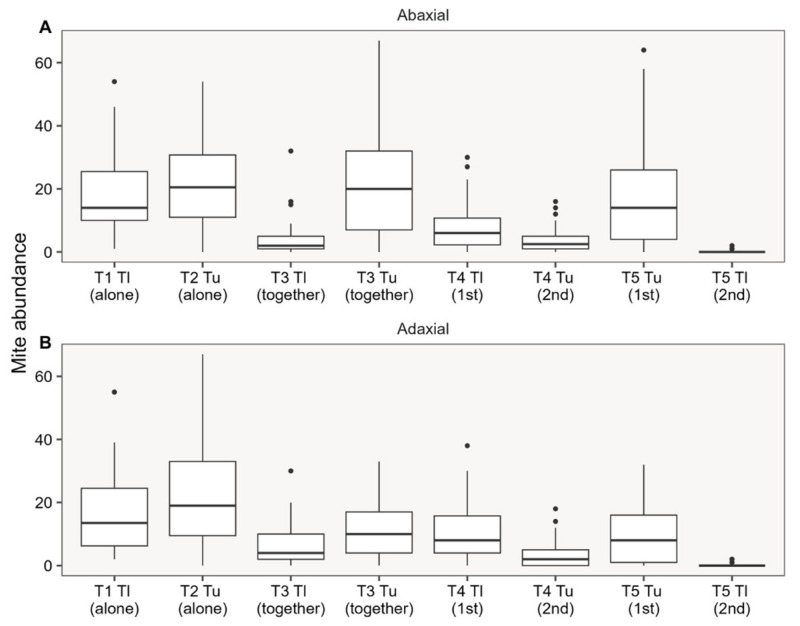
Abundance of *Tetranychus ludeni* and *Tetranychus urticae* released individually, simultaneously, or after four days on bean plants and their ability to colonize the abaxial (**A**) and adaxial (**B**) leaf regions. Notes = boxplots: box − interquartile range (IQR) covering the 2nd and 3rd quartiles, inner line = median, whiskers = data dispersion, outliers = black points. There were no significant differences when comparing the ‘abaxial’ vs. ‘adaxial’ foliar sides (for details see Table 1). (T1: *T. ludeni* alone; T2: *T. urticae* alone; T3: *T. ludeni* and *T. urticae* together; T4: *T. ludeni* (1st) and *T. urticae* (2nd); T5: *T. urticae* (1st) and *T. ludeni* (2nd)).

We did not find a significant effect on the distribution of mite populations when comparing the abaxial/adaxial sides of the leaves (χ^2^ = 2.79; df = 1; *p* = 0.09; Table 1), demonstrating that there is no distribution preference for the species evaluated in this experiment (Figure 2).

**Figure 3:**
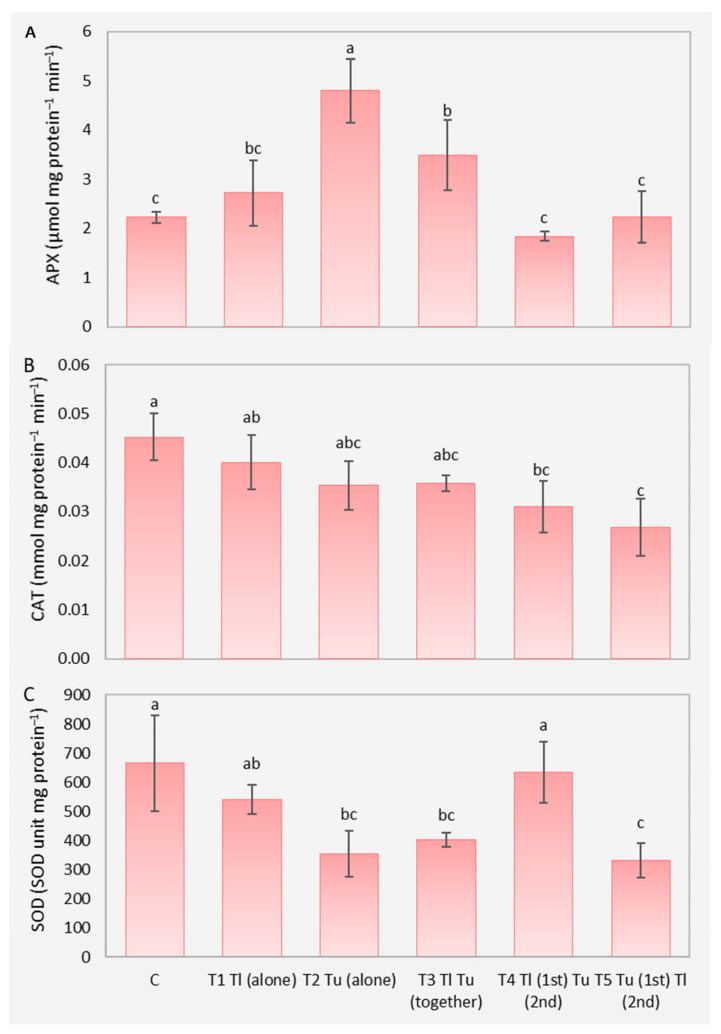
Activity of the antioxidant enzymes Ascorbate peroxidase (**A**), Catalase (**B**), and Superoxide dismutase (**C**) in bean plants infested with *Tetranychus ludeni* and *Tetranychus urticae* after 14 days. Different letters indicate significant difference between treatments. (C: Control; T1: *T. ludeni* alone; T2: *T. urticae* alone; T3: *T. ludeni* and *T. urticae* together; T4: *T. ludeni* (1st) and *T. urticae* (2nd); T5: *T. urticae* (1st) and *T. ludeni* (2nd)).

Antioxidant enzymatic activities demonstrated a significant effect of the presence of mites on APX activity (F = 17.47, *p* < 0.05), CAT (F = 7.18, *p* < 0.05), and SOD (F = 9.95, *p* < 0.05). In APX, there was an increase in enzymatic activity in T2, followed by T3 and T1, and the lowest activity was recorded PL, T5, and T4 (Figure 3A). For CAT, an increase in enzymatic activity can be observed in PL, but it is not statistically different from T1, T2, and T3. The lowest activity was found in T5, T2, T3, and T4 (Figure 3B). For SOD, the greatest increase in activity was found in PL, T4, and T1, and the lowest activity was recorded in T5, T2, and T3 (Figure 3C).

**Figure 4:**
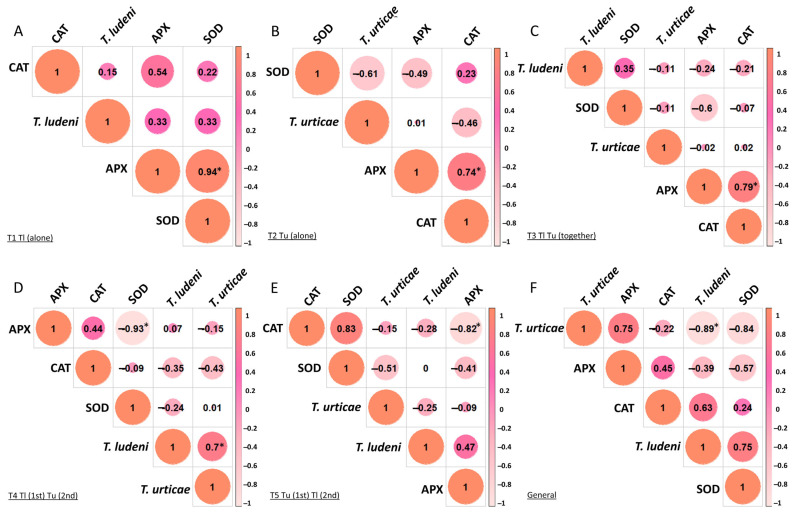
Pearson correlation coefficients of the number of mites and the activities of antioxidant enzymes of bean plants infested with *Tetranychus ludeni* and *Tetranychus urticae*, after infestation of 14 days. (**A**) T1: *T. ludeni* alone; (**B**) T2: *T. urticae* alone; (**C**) T3: *T. ludeni* and *T. urticae* together; (**D**) T4: *T. ludeni* (1st) and *T. urticae* (2nd); (**E**) T5: *T. urticae* (1st) and *T. ludeni* (2nd); (**F**) General (overall experiment average). Notes = * Asterisks indicate statistical significance (*p* < 0.05).

*Tetranychus ludeni*, in T1, was positively correlated with APX and SOD (Figure 4A). *Tetranychus urticae*, on the other hand, showed negative correlations with CAT and SOD in T2 (Figure 4B), with CAT in T4 (Figure 4D), and SOD in T5 (Figure 4E). When both species are together (Figure 4C), *T. ludeni* continues to be associated with an increase in SOD activity, while showing a negative relationship with APX, suggesting that its presence differentially stimulates specific components of the plant’s antioxidant system. In general, *T. ludeni* correlated positively with CAT and SOD and negatively with APX (Figure 4F). *Tetranychus urticae* had an inverse response, with a positive correlation with APX and a negative correlation with CAT and SOD.

## 4. Discussion

Our initial expectation was that *T. urticae*, arriving first on the plant, would harm *T. ludeni* was confirmed. The results demonstrated that the *T. ludeni* population develops better when it is alone on the bean plant. Antioxidant enzymes indicated a change in the plant defense system in relation to both species and the presence of *T. ludeni* increased the activity of CAT and SOD, while *T. urticae* increased APX. We found no preference for leaf region when both species are present on the same plant.

The results found in this study demonstrate the importance of the moment and colonization priority of species on the plant, which influenced the colonization dynamics, especially of *T. ludeni*. The early establishment of *T. urticae* hampered the subsequent colonization of *T. ludeni* in bean plants. The first colonizers of plants can inhibit the growth and/or reduce the performance of species that arrive later, especially species that occupy similar niches [41,42,43,44].

When both species arrive at the same time or when *T. urticae* (superior competitor) arrives first, the probability of exclusion of *T. ludeni* is greater. The coexistence of the two species occurred when *T. ludeni* (inferior competitor) was the first to colonize the plant. *Tetranychus urticae* demonstrated greater competitive capacity and higher population growth rate, excluding *T. ludeni* when it colonizes the plant first.

There is no information about the population dynamics and behavior of these two species when together. However, it is known that they can occur in the same plant species in the field, both in soybean and tomato [10,11,45]. The advantage of the early arrival of *T. ludeni* on the plant may be associated with a reduction in interspecific competition with *T. urticae*, and this may be one of the factors that promote the coexistence of the two species, similar to what happens with *T. urticae* and *T. evansi* [6].

There was no preference of the mites for any specific region of the leaf, possibly due to the presence of only one plant per pot, which limits the leaf options for moving the species. Fadini et al. [46] observed that *T. urticae* preferred the abaxial side, while *Oligonychus ilicis* McGregor opted for the adaxial side of strawberry leaves. These leaf colonization characteristics enabled the coexistence of the two species on the same plant, which may be related to the selection of different feeding sites. However, even if the species can coexist on the same plant, the development and permanence of tetranychid species on the same leaf surface is unlikely [47].

This is the first work that relates the coexistence of these mite species with the antioxidant defense metabolism of bean plants. In addition to the monopolization of resources, the coexistence of species can result in the induction of systemic defenses, or facilitating effects such as the negative regulation of defenses [6]. These defenses are induced through arthropod-plant coevolution relationships, in which the plant develops morphological, biochemical and behavioral characteristics to face pest attacks [48]. Among these defenses are antioxidant enzymes, responsible for maintaining redox homeostasis in cells, preventing the occurrence of oxidative stress, and consequently cellular and molecular damage to DNA, which can culminate in cell death [21,49,50].

Increased levels of APX activity in plants infested by *T. urticae* may induce plant defense by this species. However, contrary to expectations, plants that were first infested with *T. urticae* and then with *T. ludeni* did not show increased enzymatic activity. This event may have been due to suppression by *T. ludeni*, or even due to the lower production of ROS, such as hydrogen peroxide in plants infested with both species, compared to those infested only with *T. urticae*. This would require less activity of the antioxidant enzyme, justifying our findings, since this enzyme is responsible for controlling the accumulation of H_2_O_2_ and helping to mitigate oxidative damage [51]. Furthermore, it plays a crucial role in the defense mechanism against environmental stressors and pathogens [52]. Previous studies have also demonstrated increases in APX enzyme activity in cucumber plants (*Cucumis sativus* L.) and *Arabidopsis thaliana* (L.) infested with *T. urticae* [53,54].

For CAT, the highest activity was found in control plants, followed by T1, T2, and T3 plants. This enzyme eliminates H_2_O_2_ at high cellular concentrations [24,55,56]. Activities remained low in virtually all treatments, which could be attributed to other substances, both enzymatic, such as APX itself, and non-enzymatic, which remove most of the H_2_O_2_ and may have had sufficient activity to maintain ROS at basal levels, ensuring plant redox homeostasis. Other authors also found decreases in CAT activity in bean plants infested with *T. urticae* [57]. Spatio-temporal changes were listed as a possible justification for the reduction in the activity of this enzyme in plants infested by mites [27], response also found in other plant species such as *Arabidopsis* [53], *Ocimum basilicum* L. [58,59], and corn (*Zea mays* L.) [60]. A better understanding of plant antioxidant metabolism requires further studies with plants infested with other species, such as *T. ludeni*, as well as evaluating other coexistence interactions.

SOD activity, considered the front line in plant defense, was greater in control, T1 and T4. Thus, it can be suggested that *T. urticae* was responsible for reducing enzymatic activity, when alone in the plant, when together or released before *T. ludeni*. SOD is a crucial enzyme in cellular defense, as it directly regulates O_2_^·−^ and H_2_O_2_ levels, mainly by dismuting O_2_^·–^ [21,61], contributing to redox balance and protecting cells against oxidative damage [62]. SOD activity may vary due to the moment in which the plant is evaluated, as it is a metallic enzyme and is the first to be activated in plants in response to stressful factors, regardless of the intensity of the infestation [25,26,27].

Therefore, it is possible that plants infested with *T. urticae* could have had the activity of the SOD enzyme previously increased, but at the time of evaluation its intensity had already decreased. This corroborates the greater APX activity, since SOD dismutated O_2_^·–^ into H_2_O_2_, and at that moment these H_2_O_2_ levels needed to be balanced by decomposition into water through the action of APX. Other studies demonstrated that soybean cultivars infested with *T. urticae* did not present significant differences in SOD activity and, furthermore, the mite population level did not have a significant effect on the activity of this enzyme [63].

Wurlitzer et al. [15] reported the occurrence of moderate stress, characterized by an antioxidant burst of APX and CAT in the initial phase of soybean infestation by *T. ludeni*, followed by a peak in H_2_O_2_ content, resulting in a cumulative oxidative effect, evidenced by lipid peroxidation, indicating an efficient defense and signaling mechanism of the plant.

It is important to highlight that SOD activity has already been compared in conditions of isolated and combined stress (abiotic factors), with some responses observing increased activity in the combined condition [64,65]. Plant responses to two or more stressors are unique and condition-specific, including positive, neutral, or negative responses [66,67,68,69], so, in addition to the effect of the mite species (*T. ludeni* and *T. urticae*), the plant response to different interactions must be considered.

## 5. Conclusions

This study investigated the effect of the order of colonization by *T. ludeni* and *T. urticae* on bean plants, including analysis of antioxidant enzymes. The results indicated that the arrival sequence of species can be crucial in determining their final populations, influencing their interactions and coexistence. Furthermore, plants infested by *T. ludeni* and *T. urticae* demonstrated significant changes in antioxidant enzymes such as APX, CAT, and SOD. These findings offer important insights into the response of bean plants to infestation by these mites, highlighting the role of antioxidant enzymes in plant defense. The importance of new research that investigates more antioxidant enzymes and genetic aspects is highlighted, especially in the case of *T. ludeni*, which lacks information about its physiological performance in the plant.

## Figures and Tables

**Table 1:** Result of the generalized linear mixed model (GLMM), using negative binomial regression evaluating the colonization and leaf region of *Tetranychus ludeni* and *Tetranychus urticae* released individually, simultaneously or after four days on bean plants (T1: *T. ludeni* alone; T2: *T. urticae* alone; T3: *T. ludeni* and *T. urticae* together; T4: *T. ludeni* (1st) and *T. urticae* (2st); T5: *T. urticae* (1st) and *T. ludeni* (2st)). Notes = *** Asterisks indicate statistical significance (*p* < 0.001).

Fixed Effects	Χ^2^	DF	*p*-Value	
Treatments	320.94	7	<0.001 ***	
Foliar region	2.79	1	0.09	
Pairwise comparisons	Estimate	Std. error	z-value	*p*-value
T1 *T. ludeni*—T2 *T. urticae*	−0.050	0.07	−0.70	0.484
T1 *T. ludeni*—T3 *T. ludeni*	0.802	0.10	8.34	<0.001
T1 *T. ludeni*—T4 *T. ludeni*	0.384	0.08	4.73	<0.001
T1 *T. ludeni*—T5 *T. ludeni*	3.078	0.25	12.51	<0.001
T3 *T. ludeni*—T4 *T. ludeni*	−0.418	0.10	−4.03	<0.001
T3 *T. ludeni*—T5 *T. ludeni*	2.276	0.25	8.93	<0.001
T4 *T. ludeni*—T5 *T. ludeni*	2.694	0.25	10.80	<0.001
T2 *T. urticae*—T3 *T. urticae*	0.187	0.08	2.40	0.020
T2 *T. urticae*—T4 *T. urticae*	1.006	0.10	10.18	<0.001
T2 *T. urticae*—T5 *T. urticae*	0.319	0.08	4.03	<0.001
T3 *T. urticae*—T4 *T. urticae*	0.820	0.10	7.96	<0.001
T3 *T. urticae*—T5 *T. urticae*	0.132	0.08	1.57	0.131
T4 *T. urticae*—T5 *T. urticae*	−0.688	0.10	−6.62	<0.001
Random effect	Variance	Std. dev.	*N*	
Plants	0.010	0.010	10	
Leaves	0.043	0.020	5	
Model selection	AICc ^1^	dAICc ^2^	DF ^3^	Weight ^4^
GLMM negative binomial (selected)	2465	0.0	12	1
GLMM Poisson	7436	4970	11	<0.001

Std. Error: Standard Error; Std. dev: Standard Deviation; z-value: Standard score, i.e., standard deviations from their means. Negative values when raw score is below the mean, positive when above; *p*-value: probability to find z-scores by chance. ^1^—computation of AIC; ^2^—differences among AICs; ^3^ DF—degree of freedom; ^4^—weight of AICs.

## Data Availability

The original contributions presented in the study are included in the article. Further in-quiries can be directed to the corresponding author.
